# A Memoir on Amputation of the Thigh at the Hip-Joint (with a Successful Case)

**Published:** 1846-07

**Authors:** 


### Art. IX.

*A Memoir on Amputation of the Thigh at the Hip-Joint (with a successful Case).* By William Sands Cox, f.r.s.—*London,* 1846. Folio, pp. 47.

The present work answers very well to its title; it is indeed a very complete account of the operation of amputation at the hip-joint. The history of the operation from the period when it was first proposed, down to the latest date, is minutely drawn up from the materials collected by Hedenus and Velpeau, which are added to by Mr. Cox's research; and it is right to add that Mr. Cox has evidently in many, indeed, we believe most instances, consulted the original authorities referred to by Hedenus and Velpeau, and in one or two instances corrected the statements of the former writer. In the account of the various methods of operating which have been either proposed or executed, Mr. Cox does not, indeed, go into needless details respecting the minute steps of many of the modifications which have been contrived of the three leading methods, the circular, the flap, and the oval operations; but he describes each of those three classes of operations with all necessary explicitness and clearness. The method which Mr. Cox seems to consider as in itself the best, and which he adopted in the case that gave occasion for the publication of the present memoir, is the following:

"The patient is to be supported in a recumbent posture, and so placed that the tuberosities of the ischia project a little beyond the margin of the table. The compressor (Segnoroni's) is to be applied over the external iliac artery as it passes over the body of the os pubis, and the thigh must be partially flexed. The operator is to stand on the other side; the point of a narrow double-edged knife, about twelve inches in length, is to be boldly and steadily introduced at an inch below the anterior-superior spinous process of the ilium, and made to glide across the neck of the femur, parallel with, and a little below Poupart's ligament, so as to pass beneath the muscles of the anterior and internal region of the thigh, and beneath the superficial and deep femoral vessels, and be brought out about an inch below the margin of the anus. The knife is now to be carried downwards in a line with the anterior surface of the femur, from three to three inches and a half, in proportion to the bulk of the limb, and then brought obliquely downwards and forwards through the integuments, by which means the anterior flap will be formed. The flap is immediately to be thrown back, and its vessels, viz. the deep and superficial femoral arteries, at once compressed.

"The limb is now to be depressed and rotated outwards, when the capsule, being fairly exposed, is to be divided close to the edge of the acetabulum, and next the round ligament. The head of the bone will then be made to slip from the socket by a slight rotatory movement outwards, and the knife is to be carried through the joint. The posterior layer of the capsule, the extensor muscles of the hip attached to the trochanter major, and the small rotators inserted into the digital fossa of the same process, are then to be divided; the knife is to be passed downwards on a line with, and close to the posterior surface of the femur for about three inches or three inches and a half, and afterwards downwards and backwards through the integuments—thus completing the operation. The posterior vessels are first to be secured, and afterwards the vessels of the anterior region. The majority of surgeons recommend that the synovial membrane and cartilage of the acetabulum shall be scraped off; but I believe this to be a bad practice, and often productive of suppuration and disease of the bone. The cartilage and synovial membrane will take on adhesive inflammation in the same manner as the other soft parts, and the secretion will be suspended. The nerves should be cut off as high as possible; for, if they should become engaged in the cicatrix of the wound, they would cause the most violent pain, not only during the cure, but after the wound was healed." (pp. 27-9.)

This operation, Mr. Cox says, "is a modification of Béclard's and Lisfranc's plan," and it combines some features of both methods, improved perhaps by their fusion. As a general method it is, we have no doubt, a good one and it answered exceedingly well in Mr. Cox's case; the limb, we are told, having been removed within thirty-five seconds. Of course Mr. Cox does not mean to recommend this method in every case; for, though we do not perceive that he has adverted to the point, in some instances the plan of operation must be governed by the direction in which the injury or disease for which it is performed has extended; and a method not advisable in itself may be the only one left for the operator to adopt. "The great objections," Mr. Cox observes, "to amputation at the hip-joint have been the difficulty of restraining the hemorrhage, and the shock given to the nervous system by the operation itself." (p. 25.) The rapidity with which the operation he proposes may be performed, tends greatly to obviate the latter objection, and the former he thinks is overcome by "the invention of the arterial compressor by Dr. Segnoroni, of Padua." We confess that we would feel more confidence in having the artery commanded by the hands of a good assistant than in trusting to the compressor; it would be absurd to question that the latter may be most effectual, but we would prefer the intelligent machine to the merely mechanical apparatus. Mr. Cox in describing Larrey's operation mentions his practice of tying the main artery of the limb as closely as possible to Poupart's ligament, but does not think it necessary to occupy space in condemning the proceeding. We may, however, mention that if the surgeon trusts to this method as a certain guarantee against hemorrhage, he may find it perfectly illusory. Thus in the operations performed by Peliken and Korseniewsko the vessel was tied, but nevertheless the hemorrhage was so profuse as apparently to have materially contributed to the fatal result of those operations.

Mr. Cox gives two statistical tables of the result of the operations in which amputation at the hip-joint has been performed, one exhibiting the successful, the other the unsuccessful cases. The whole number of cases contained in both tables amounts to 84, of which 26 were successful and 58 unsuccessful. These tables are not only most diligently and faithfully compiled, but, as far as our knowledge extends, almost complete. We are indeed aware of but one omission, which is a case, mentioned by Velpeau as having been seen by Delaunay at Moscow, in which the patient had perfectly recovered after disarticulation of the thigh, performed in consequence of gangrene of the limb. It might perhaps be objected that this case rests on hearsay, but it is in this respect on a parity with the case of the English sailor operated on after the battle of Aboukir. This case increases the number of successful operations to 27, and the entire number of operations recorded to 59. We think too that the case of M. Baffos, which Mr. Cox ranks among the unsuccessful cases, might perhaps be fairly placed among the successful ones, so far as the operation was concerned, for, to give Mr. Cox's summary of the result, "the wound healed healthy; on the sixty-third day pain came on, the cicatrix ulcerated and opened, and he died at the end of the third month." Death resulted in this case from constitutional causes not from the results of the operation itself, and, therefore, in appreciating merely the results of the operation we should be rather inclined to place it among those in which the *operation* succeeded. Mr. Cox, however, states the facts accurately so that every one can form their own opinion, and is probably right in rejecting from his table of successful cases a case open to any objection.

We availed ourselves of the materials collected by Mr. Cox to examine whether any connexion could be traced between the success or failure of the operation and the cause for which it was performed, but the latter particular is unspecified in so many cases that no satisfactory conclusion can be deduced. It may, however, be mentioned that of the successful operations 14 were performed for traumatic causes; 7 (including the case mentioned by Delaunay) in consequence of disease; while in 6 the nature of the affection under which the patient laboured is unknown. Of the unsuccessful operations 20 were performed for injuries, 18 for disease, and 20 for causes not mentioned.

The disease under which Mr. Cox's patient laboured was one of an unusual character. A young woman, aged 23, had undergone amputation of the thigh, at the age of 14, in consequence of disease of the knee-joint. This, therefore, we may observe, is the third case in which disarticulation of the hip has been performed after previous amputation of the limb above the knee, and all three operations were successful. About three months after this first operation ulcers formed round the cicatrix, and a very painful "substance" formed at the back of the stump; the ulcers healed partially but never completely, from the centre towards the circumference. About six years after the operation the integuments became hard and thick, and at length fungous growths formed on the surface, aud the parts became affected with gradually increasing pain. A variety of treatment was employed during the course of several years, but the disease made progress and on July 1st 1844—

"The integuments extending upwards, anteriorly for about three inches, and posteriorly for about four inches and a half, are of a dull white colour, and of a cartilaginous hardness; and above this, for a limited extent, anteriorly, posteriorly, and laterally, the same parts have a glazed, corrugated appearance, like that presented by an old cicatrix. Patches of fungous growth, of a livid colour, protrude from one half to one third of an inch from the general surface, at intervals. From these excrescences blood occasionally exudes and at times a sanious fluid. These excrescences are extremely tender and bleed on the least touch. The integuments of the upper part of the stump are of a perfectly healthy character and feeling. There is no enlargement of the cutaneous veins, or of the inguinal or femoral glands. The stump generally is tender to the touch; the pain nearly constant, sometimes of a dull aching character, sometimes throbbing." (pp. 30-1.)

The character and general appearance, and health of the patient were perfectly satisfactory. On examining the parts after the operation, the integuments were found to be converted into a mass of cartilaginous hardness, pearly white, and from three eighths to five eighths of an inch thick. Under the microscope this structure appeared cellular and vascular, with myriads of minute globules, interspersed with spindle-shaped bodies, as observed by Müller in many tumours. The adipose tissue was very dense and intersected with fibrous bands, as were also the muscles, which had a granular appearance, were softened, and had undergone fatty degeneration, with the exception of those inserted into the trochanters. The arteries were not enlarged, and the muscular and perforating branches when injected, were found to terminate in a vascular network distributed to the integuments and fungoid growth. The nerves terminated in bulbous enlargements, that of the great sciatic as large as a walnut, grayish, solid, vascular, and sending off fibrous bands which were lost in the adipose tissue. The bone was sound. It would be tedious to trace the progress of the case after the operation, suffice it to say that after three months the patient was discharged cured.

There are one or two things respecting Mr. Cox's memoir of which we must complain. One is the cumbrous and expensive form in which it is published. Surely, if Mr. Cox did not wish it to appear in the transactions of some society, such as the Medico-Chirurgical or Provincial, he might at least have brought it out in the ordinary size, and at the ordinary expense of an octavo pamphlet. Neither can we pass without comment, the plan of publishing the work by subscription adopted by the author. The benevolent and very laudable desire of Mr. Cox "to benefit the poor patient," might have been carried into effect as well or better, without the necessity of offering to the subscribers the bonus of a *Memoir on Amputation of the Thigh.* What do the Prince Albert, the Earl Howe, and the scores of reverend and other gentry care for such an offering? The few medical men in the list might, indeed, have been properly presented with copies; but all the others could and would very well have gone without, while the money expended on the copies provided for so large a number of non-professional men, would have added not a little to the fund "for the benefit of the poor patient;" and Mr. Cox would have escaped the imputation to which we know he has rendered himself (we believe innocently) obnoxious, of having, in this proceeding, contemplated a novel and most effective method of puffing and glorifying himself, in the eyes of his non-professional friends. It is clearly for the non-professionals that the full-length portrait of pretty Elizabeth Powis has been prefixed as a frontispiece; as it is calculated to convey no tittle of information to surgeons; while the engraving, and especially the colouring of it, in red, pink, blue, green, and yellow, must have made a large hole in the treasure-trove of the "poor patient." All this is certainly in the worst possible taste: it more resembles the proceeding of one of our vain neighbours across the channel, than that of an English surgeon of high standing in his profession; and is totally unworthy of Mr. Cox's acknowledged reputation as a man of science and a surgical authority.

In its present garb comparatively few are likely to become acquainted with this memoir. Either of the mediums we have suggested, particularly the first, would have given it a greatly more extended circulation; and we wish, for the profession's sake as well as the author's, that it had been rendered more generally accessible, as we know nowhere else that the mere English reader could find satisfactory information respecting the results of this particular operation. Indeed, we are not acquainted with any work on this subject in any language which contains that information so fully.

